# Therapeutic effect of various ginsenosides on rheumatoid arthritis

**DOI:** 10.1186/s12906-021-03302-5

**Published:** 2021-05-25

**Authors:** Meng Zhang, Hongwei Ren, Kun Li, Shengsheng Xie, Ru Zhang, Longlong Zhang, Jiaxuan Xia, Xing Chen, Xilin Li, Jianxin Wang

**Affiliations:** 1 School of Pharmacy, Shanghai University of Traditional Chinese Medicine, Shanghai, 201203 China; 2 Department of Pharmaceutics, School of Pharmacy, Fudan University & Key Laboratory of Smart Drug Delivery, Ministry of Education, Shanghai, 201203 China; 3 Science and Technology Innovation Center, Guangzhou University of Chinese Medicine, Guangzhou, 510405 China; 4 Institute of Integrative Medicine, Fudan University, Shanghai, 201203 China

**Keywords:** CIA, Ginsenoside compound K, *Panax ginseng*, Rheumatoid arthritis, Therapeutic effect

## Abstract

**Background:**

Rheumatoid arthritis (RA) is an autoimmune disease which causes disability and threatens the health of humans. Therefore, it is of great significance to seek novel effective drugs for RA. It has been reported that various ginsenoside monomers are able to treat RA. However, it is still unclear which ginsenoside is the most effective and has the potential to be developed into an anti-RA drug.

**Methods:**

The ginsenosides, including Rg1, Rg3, Rg5, Rb1, Rh2 and CK, were evaluated and compared for their therapeutic effect on RA. In *in vitro* cell studies, methotrexate (MTX) and 0.05% dimethyl sulfoxide (DMSO) was set as a positive control group and a negative control group, respectively. LPS-induced RAW264.7 cells and TNF-α-induced HUVEC cells were cultured with MTX, DMSO and six ginsenosides, respectively. Cell proliferation was analyzed by MTT assay and cell apoptosis was carried out by flow cytometry. CIA mice model was developed to evaluate the therapeutic efficacy of ginsenosides. The analysis of histology, immunohistochemistry, flow cytometry and cytokine detections of the joint tissues were performed to elucidate the action mechanisms of ginsenosides.

**Results:**

All six ginsenosides showed good therapeutic effect on acute arthritis compared with the negative control group, Ginsenoside CK provided the most effective treatment ability. It could significantly inhibit the proliferation and promote the apoptosis of RAW 264.7 and HUVEC cells, and substantially reduce the swelling, redness, functional impairment of joints and the pathological changes of CIA mice. Meanwhile, CK could increase CD8 + T cell to down-regulate the immune response, decrease the number of activated CD4 + T cell and proinflammatory M1-macrophages, thus resulting in the inhibition of the secretion of proinflammatory cytokine such as TNF-α and IL-6.

**Conclusion:**

Ginsenoside CK was proved to be a most potential candidate among the tested ginsenosides for the treatment of RA, with a strong anti-inflammation and immune modulating capabilities.

## Background

Rheumatoid arthritis (RA) is one of the most prevalent chronic autoimmune diseases, characterized by joint destruction, synovium inflammation as well as vascular proliferation [1]. The incidence of RA ranges from 0.5 to 1% worldwide, within women and the elderly accounting for the majority [2, 3]. RA without effectively control can reduce the quality of life and even may lead patients to disability.

Currently, the drugs for RA treatment include non-steroidal anti-inflammatory drugs, glucocorticoids, and disease-modifying antirheumatic drugs (DMARDs) [4]. Based on the origin, DMARDs can be classified into conventional synthetic drugs, biological drugs and biosimilars and targeted syntheticts, such as Janus kinase (Jak) inhibitors tofacitinib [4–7].The leading DMARD is methotrexate (MTX), which has been widely used to treat RA in clinic. Biological agents are used when arthritis is uncontrolled or toxic effects arise with DMARDs [8, 9]. Although there are many drugs available, the therapeutic effect of the drug in many patients is still unsatisfactory. Moreover, anti-RA drugs are usually restricted by their severe side effects, accompanied by increased risk of osteoporosis [10], gastric mucosa damage [11], metabolic disorders and secondary infections [12]. Therefore, a novel treatment approach with ideal curative effect and low toxicity is urgently needed.

In recent years, using traditional herbs to treat chronic diseases has become popular. *Panax ginseng Meyer*, pertaining to the *Panax ginseng* family, has been widely used as a traditional Chinese medicine for thousands of years [13]. The main active ingredients of ginseng are ginsenosides, which have been reported as having a variety of pharmacological effects, including the treatment of cardiovascular and cerebrovascular diseases [14, 15], protective effect on salivary dysfunction [16], anti-inflammatory [17, 18], immunomodulation [19], and so on.

Based on the structure, the dammarane type of ginsenoside can be sorted into two types: ginseng diol-type A, glycogen 20 (S) -original ginseng diol, and ginseng triol-type B, aglycones for 20 (S) - original ginseng triol. The former includes the majority of ginsenoside monomers, such as ginsenoside Rb1, Rc, Rh2, Rg3, Rg5, and the latter contains ginsenoside Re, Rg1, and so on [14]. Besides, ginsenoside CK is a metabolite in the intestinal tract of natural diol ginsenosides.

Various ginsenoside monomers have been reported to be effective in the treatment of RA. For instance, Chen et al. [20] concluded that ginsenoside CK down-regulates the percentage of activated T cells and up-regulates the expression of naive T cells and Treg cells in the spleen to relieve autoimmune arthritis. It may also rely on T cells to down-regulate memory B cells to play an anti-inflammatory effect [21] . Kim et al. [22] and Wang et al. [23] obtained the results that ginsenoside Rb1 and ginsenoside CK can inhibit TNF-α upregulation in peripheral blood mononuclear cells, fibroblast-like synoviocytes and chondrocytes as well reduces cell infiltration and cartilage destruction in the arthritic joint. Zhang et al. [24] found that ginsenoside Rg1 ameliorates adjuvant arthritis through increasing PPAR- γ protein expression, inhibiting IĸBα phosphorylation and NF- ĸB nuclear translocation.

**Fig. 1:**
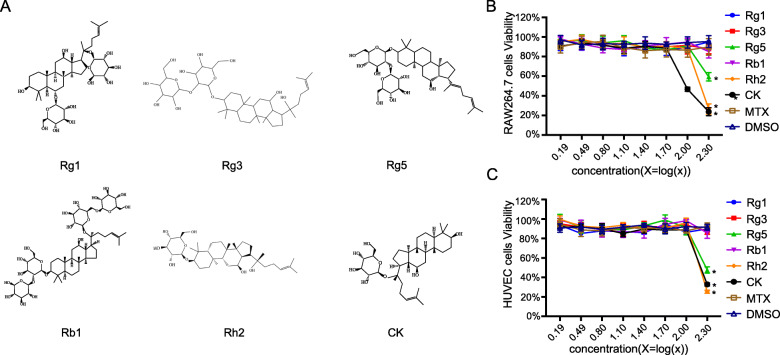
The chemical structures of six ginsenoside monomers (**a**). The MTT assay results of six ginsenosides on the viability of RAW 264.7 cells (**b**) and HUVEC cells (**c**) as a function of concentrations for 24 h. DMSO and MTX was served as a negative group and a non-ginsenoside positive control group, respectively. Data were presented as the mean ± SD (*n* = 6). *p < 0.05, vs DMSO group

Although many studies have reported the promising therapeutic effect of ginsenosides for RA, no ginsenoside has been approved as an anti-RA drug since their effect and action mechanisms are not elucidated clearly. Since most of the studies were carried out in different labs, the results are not comparable. It is still unknown which ginsenoside has the best therapeutic effect and is mostly potential to be developed further. Thus, in this study, in the light of the structure and pharmacological activities, six ginsenoside monomers (Fig. 1a), Rg1, Rg3, Rg5, Rb1, Rh2 and CK, were chosen to compare and evaluate on the basis of their therapeutic effect on RA in vitro and in vivo.

## Methods

### Reagents and drugs

Ginsenoside Rg1, Rg3, Rg5, Rb1, Rh2, CK (purity > 98% as determined by HPLC) and methotrexate were purchased from Dalian Meilun Biotech Co., Ltd. (Dalian, China). 3-[4,5-dimethyl-2-thiazolyl]-2,5-diphenyl-2H-tetrazolium bromide (MTT), tumor necrosis factor (TNF-α), lipopolysaccharide (LPS), Type IV Collagenase were provided by Sigma-Aldrich Company (St. Louis, MO, USA). Propidium iodide (PI) and Annexin V-FITC were acquired from BioVision Inc. (Milpitas, CA, USA). Complete Freund’s adjuvant (CFA) and Type II bovine collagen were obtained from Chondrex, Inc. (Redmond, WA, USA). Mouse antibody CD45-Percp-Cyanine5.5, CD4-FITC, CD8-PE-Cy7, F4/80-FITC, CD86-PE were purchased from BioLegend Co, Ltd. (San Diego, CA, USA). TNF-α and the IL-6 ELISA Kit were gained from Thermo Fisher Scientific (Shanghai, China).

### Research timeline

The first to the third month:Cell experiments were conducted, including cell culture, cytotoxicity of ginsenosides experiments, cell proliferation assay and cell apoptosis experiment.

The fourth to the twelfth month:Animal experiments were carried out, including feeding mice, establishing CIA models, treating CIA mice with ginsenosides and evaluating arthritis index, analyzing histopathology, immunohistochemistry and immune cells by flow cytometry, detecting cytokines and evaluating safety.

### Cytotoxicity of ginsenosides

RAW264.7 cells (Mouse leukemia cells of monocyte macrophage) and HUVEC cells (Human umbilical vein endothelial cells) were acquired from Cell Bank at the Chinese Academy of Sciences (Shanghai, China). The cells were cultured in DMEM medium supplemented with 10% fetal bovine serum, 1% penicillin-streptomycin in an incubator with 5% CO_2_ at 37 °C.

MTX and six ginsenoside powders with 98% purity were dissolved in DMSO respectively and then diluted to the required concentration with serum-free medium. The final concentration of DMSO was 0.05%, which is not harmful to the cell viability. DMSO and MTX was set as a negative control and a non-ginsenoside positive control, respectively.

### Cell proliferation assay

MTT assays were used to estimate the effects of ginsenoside on the proliferation of LPS induced RAW264.7 cells [25] and TNF-α induced HUVEC cells. In brief, RAW264.7 cells were cultured with LPS (100 ng/ml, final concentration in the well) and HUVEC cells were cultured with TNF-α (10 ng/ml, final concentration in the well) for 24 h, followed by incubating with six ginsenosides at different concentrations. At the end of treatment, MTT solution was added into each culture media and incubated for 3 h. After that, the cell culture solution was removed and replaced by DMSO solution to dissolve formazan crystals. Each well was placed in a shaker at low speed for 10 min. Then the optical density (OD) was measured at 570 nm using the microplate reader, and the cell inhibition rate (%) was calculated according to the formula as follows:

$$ cell\kern0.5em inhibition\kern0.5em rate\kern0.5em \left(\%\right)=100\%-\frac{OD\kern0.5em \left( ginsenosides\kern0.5em group\right)\kern0.5em -\kern0.5em OD\kern0.5em \left( blank\kern0.5em wells\right)}{OD\kern0.5em \left( control\kern0.5em group\right)\kern0.5em -\kern0.5em OD\kern0.5em \left( blank\kern0.5em wells\right)} $$

### Cell apoptosis assay

The cells in each group were collected, washed with PBS, and double-stained with PI and Annexin V-FITC based on manufacturer’s instructions. After incubation at 37 °C for 10 min, flow cytometry (Beckman Coulter, Brea, CA, USA) was used to quantitatively analyze of cell apoptosis. The number of necrosis (PI+), early apoptotic cells (Annexin V +, PI-) and late apoptotic cells (Annexin V-, PI +) were measured for further analysis.

### Animals

Eight-week-old male DBA/1 mice were acquired from Shanghai SLAC Laboratory Animal Co Ltd. and maintained in the SPF Animal Laboratory of Fudan University, where water and food were available *ad libitum*. All in vivo experimental procedures were given permission by the Animal Experiment Ethical Committee of Fudan University (2017–03-YJ-SXY-01). Six mice were randomly selected as normal mice and the rest were used to prepare the CIA model.

### Induction and treatment of CIA

Type II bovine collagen was dissolved in 0.1 mol/ml acetic acid and mixed in an ice bath with an equivalent concentration of 2 mg/ml of complete Freund’s adjuvant [26]. DBA/1 mice were injected intradermally with 100 μl above-mentioned emulsion at the base of the tail twice. The first day of immunization was marked as day 0, and the second immunization injection was given on day 21 [27]. In order to avoid the possible adverse effect of DMSO and increase the stability of the solution, 0.5% Tween-80 was added as a solubilizer to fabricate ginsenoside solutions [28]. After the onset of joint swelling, the CIA mice were randomly divided into seven groups (*n* = 6): CIA + 0.5% Tween-80 as control group, CIA + Rg1, CIA + Rg3, CIA + Rg5, CIA + Rb1, CIA + Rh2 and CIA + CK as treatment groups. Treatment groups were performed by intravenous injection of each kind of ginsenoside (15 mg/kg) once every 2 days for 15 times. The healthy mice without the administration of Freund’s adjuvant were given with equal PBS and set as normal group.

### Arthritis assessment

The typical characteristic of the occurrence and development of RA is the degree of joint swelling. After the onset of arthritis, CIA mice were evaluated every 2 days by two independent observers who were blinded to treatment pattern. The evaluated rules are as follows: normal appearance, scored 0; paws with swelling of one finger joint, scored 1; paws with swelling of two or more finger joints but not the entire paw, scored 2; moderate swelling and redness in three joints or extend to the entire paw, scored 3; severe inflammatory and swelling reaction to the entire paw and ankle, scored 4 [29]. Four paws were scored separately, with each animal’s limbs score adding up to a maximum of 16. The score of each mouse’s paws were accumulated as an arthritis index. Typically, an arthritis score of 4 or more indicates that the CIA has been favorably simulated [30]. At the end of the experiment, joint tissues and organs were collected after the mice were euthanized in the form of cervical dislocation.

### Histological analysis

The ankle joints of each group were skinned and fixed in formalin for 48 h, then decalcified in 15% EDTA for 1 month. After decalcification, the tissues were embedded in paraffin and sectioned at a thickness of 4 μm. Sections were stained with Hematoxylin and Eosin (H&E) to reveal the changes of joint tissue structure.

### Immunohistochemistry study

Tissue sections in all groups were deparaffined before incubation with specific primary antibodies (TNF-α and IL-6), and then appropriate secondary antibodies were added after antigen recovery and serum sealing. The nuclei of osteoblasts were stained yellow or brown revealed TNF-α or IL-6 positive immunocytochemical reaction. Image-pro plus 6.0 (Media Cybernetics, Inc., Rockville, MD, USA) Image analysis software was used to measure the cumulative optical density (IOD) of TNF-α and IL-6 positive expression and the pixel area of the tissue, then the mean density (IOD/AREA) was calculated.

### Flow cytometric analysis

After peeled and cut, the mice joints were incubated with a mixture of collagenase and DNA enzyme for 30 min (37 °C) and then homogenized via centrifugation at 3000 rpm for 10 min (4 °C), precipitated cells were obtained and washed with PBS for 2 times before resuspended. The resuspended cells were evenly divided into 2 parts for macrophage and T cell analysis, respectively. One part of cells was added into the mouse antibody CD45-Percp-Cyanine5.5, CD4-FITC and CD8-PE-Cy7 to analyze and screen T cells. After incubation in dark for 30 min, the surplus antibodies were washed with PBS and resuspended for analysis by flow cytometer (BD Biosciences). Another cell was added with mouse antibody CD45-Percp-Cyanine5.5, F4/80-FITC, CD86-PE to label macrophages, and the operations were consistent with the above.

### Cytokine detection

The collected joints of mice were homogenized after incubating with collagenase and DNA enzyme mixture for 30 min (37 °C), the tissue supernatants were obtained after centrifuging at 3000 rpm for 10 min (4 °C) and then stored at − 80 °C refrigerator until analysis. TNF- α and IL-6 levels were analyzed using ELISA kit. All procedures were based on manufacturer’s recommendations.

### Safety evaluation

To assess the safety of ginsenosides, major organs (including the heart, liver, spleens, lung and kidneys) of the mice were collected for paraffin embedding and H&E staining. The sample were photographed with a fluorescent microscope.

### Statistical analysis

The results were expressed as mean ± standard deviation (SD) and analyzed by GraphPad Prism v5.0 software. The comparison of two groups was conducted by *t* test, and the comparison of multiple groups was conducted by one-way analysis of variance (ANOVA) followed by *Dunnett’s* test. *p* value lower than 0.05 was considered statistically significant.

## Results

### Effects of ginsenosides on the viability of RAW264.7 cells and HUVEC cells

Figure 1(b and c) showed the effects of six ginsenosides on the viability of RAW 264.7 cells and HUVEC cells as a function of concentrations. It was observed that 0.05% DMSO had a slight effect on the cell viability, but no statistically significance was found. From the results, it could be concluded that ginsenoside Rg1, Rg3, Rb1 and MTX, do not affect the activity of RAW264.7 cells or HUVEC cells up to the concentration of 200 μg/ml. When Rg5, Rh2 or CK concentration was lower than 100 μg/ml, HUVEC cells grew normally. When the concentration of ginsenoside Rh2 and Rg5 was higher than 100 μg/ml or the concentration of CK was higher than 50 μg/ml, the growth of RAW 264.7 cell was obviously inhibited. Based on the results, we further investigated the inhibitory effect of ginsenosides on the proliferation of RAW 264.7 cells and HUVEC cells, and the safe concentration of each ginsenoside was chosen, respectively.

### Effects of ginsenosides on the proliferation of RAW264.7 cells induced by LPS and HUVEC cells induced by TNF-α

**Fig. 2:**
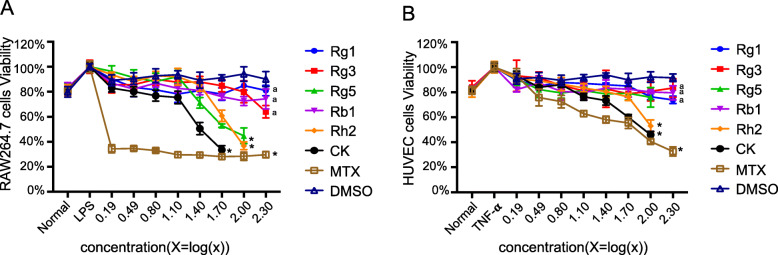
MTT assay results of six ginsenosides on the proliferation of RAW264.7 cells induced by LPS (**a**) and HUVEC cells induced by TNF-α (**b**) as a function of concentrations for 24 h. DMSO and MTX was served as a negative group and a non-ginsenoside positive control group, respectively. Data are presented as the mean ± SD (*n* = 6). *p < 0.05, vs DMSO group; ^a^*p* < 0.05, vs CK group

As shown in Fig. 2a, the proliferation ability of RAW264.7 cells increased under LPS induction. No significant inhibitory effect was observed for ginsenoside Rg1, Rg3 and Rb1 in the range of test concentration. Meanwhile, when the concentration was higher than 50 μg/ml, Rg5, Rh2 and CK exhibited certain inhibitory activity. At the concentration of 50 μg/ml, the cell survival rates of CK, Rh2 and Rg5 groups were 35.54 ± 3.29%, 60.80 ± 4.51% and 52.97 ± 1.31%, respectively. Hence, the effect of CK was found to be the strongest among six ginsenosides. Besides, the inhibition effect of Rh2 was higher than that of Rg5 when the concentration reached to 100 μg/ml.

The results in Fig. 2b showed that the inhibition effect of ginsenosides Rg1, Rg3, Rg5 and Rb1 on HUVEC cell proliferation stimulated by TNF-α was not obvious within the range of concentration. Meanwhile, Rh2 and CK could suppress the proliferation in a concentration-dependence mode. When the concentration reached to 100 μg/ml, the inhibition rate of CK and Rh2 groups was 54.06 ± 2.94% and 46.9 ± 4.83%, respectively. No effect of 0.05% DMSO was found, meanwhile, MTX, as a positive drug control, had obvious inhibitory effect on the proliferation of two kinds of cells.

### Effects of ginsenosides on the apoptosis of RAW264.7 cells and HUVEC cells

In the course of arthritis, the proliferation of vascular endothelial cells and macrophages will further lead to the formation of pannus and inflammatory response [31–33]. Therefore, promoting the apoptosis of these cells is conducive to the treatment of arthritis. In order to further study the mechanism of ginsenosides, Annexin V-FITC and PI double staining were used to evaluate their effect on cell apoptosis. Annexin V-, PI- means viable cells; Annexin V-, PI+ means necrosis cells; Annexin V+, PI- means early apoptotic cells and Annexin V+, PI+ means apoptotic cells. The RAW264.7 cells apoptosis induced by 4% paraformaldehyde and the HUVEC cells apoptosis induced by H_2_O_2_ were taken as the control group, respectively. Based on the results of the previous pharmacodynamics experiments, the concentration of ginsenosides was chosen as 50 μg/ml.

**Fig. 3:**
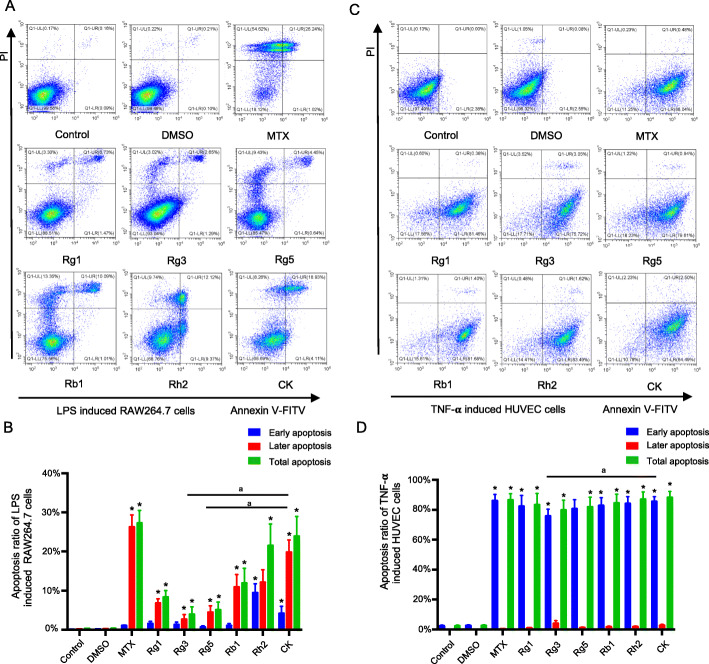
Effects of six ginsenosides on the apoptosis of RAW264.7 cells and HUVEC cells. Representative scatter plots of Annexin V/PI analysis of RAW264.7 cells (**a**) and HUVEC cells (**c**). Percentage of early apoptotic cells, late apoptotic cells and total apoptotic cells of RAW264.7 cells (**c**) and HUVEC cells (**d**) in each group. Data were presented as the mean ± SD (*n* = 3). *p < 0.05, vs DMSO group; ^a^*p* < 0.05, vs CK group

The results in Fig. 3 showed that there was no significant difference between control group and DMSO group, indicating that 0.05% DMSO had no effect on the apoptosis of cells. Ginsenosides could promote the apoptosis of two abnormal proliferation cells, compared with the DMSO group. In contrast, the power of ginsenosides inducing the apoptosis of HUVEC cells was stronger than that of RAW264.7 cells. Besides, in RAW264.7 cells, the percentage of later apoptosis induced by ginsenosides was higher than that of early apoptosis, while in HUVEC cells, by contrary, with early apoptosis accounting for a larger proportion. What’s more, throughout the whole apoptosis experiments, among six kinds of ginsenosides, Rh2 and CK showed the best apoptotic effects in both two cell lines.

### Effects of ginsenosides on arthritis development in CIA mice

**Fig. 4:**
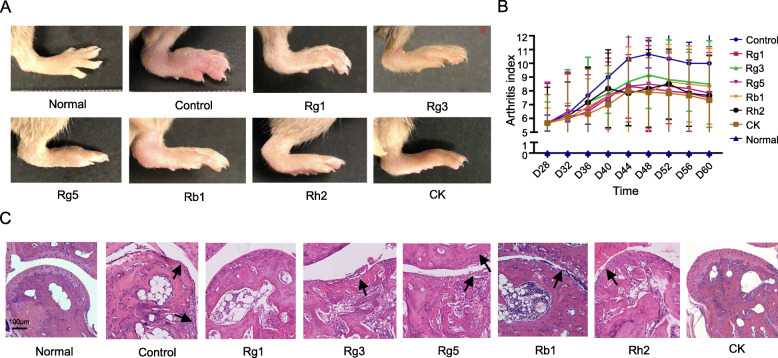
Effects of six ginsenosides on arthritis development in CIA mice after treatment. **a** The joint images of representative mice in each group at the end of administration. **b** The arthritis index treated with different ginsenoside as a function of time. Data were expressed as mean ± SD, *n* = 6. *p* < 0.05 for each ginsenoside group vs the control group. **c** Pathological sections of hematoxylin and eosin stained ankle joints in each group, the arrows indicate pannus formation and bone destruction. (Scale bar = 100 μm). PBS and 0.5% Tween was set as normal group and control group, respectively

The changes of CIA mice joint were used to evaluate the treatment effect of ginsenosides against arthritis development. Starting from the onset of arthritis, CIA mice were scored every 2 days as described previously [29]. As shown in Fig. 4a, compared with the healthy mice in normal group, the limbs of CIA mice were characterized by redness and swelling, and the arthritis index scores of the control group were significantly higher than those of normal group. On day 48, the degree of joint swelling of control mice reached to its peak. Nevertheless, started from day 30, the mice in the treatment groups were injected with 15 mg/kg of ginsenosides once every 2 days, and the mice in the control group and the normal group were given the same dose of 0.5% Tween-80 and PBS. After the treatment, ginsenosides Rg1, Rg3, Rg5, Rb1, Rh2 and CK group had an arthritis index of 7.50 ± 3.71, 8.50 ± 3.14, 7.83 ± 2.95, 8.33 ± 2.74, 7.67 ± 2.88 and 7.33 ± 3.295, respectively, while the control group had a score of 10.00 ± 2.08. Although the inflammation of the joint could not be completely inhibited, the swelling of the mice in each group were alleviated to different degrees, and the arthritis index was fell off apparently as well, suggesting that ginsenosides have therapeutic effects on arthritis. Among them, the effect of ginsenoside CK on alleviating joint swelling was stronger than that of other ginsenosides (Fig. 4b).

### Effects of ginsenosides on joint histopathology

As demonstrated in Fig. 4c, the joint of normal mice was healthy and well-structured. In the control group, the articular surface was rough and the pannus were formed. The cartilage surface was covered by fibrous tissues, which hindered the nutrient absorption of normal cartilage. In the group of Rg3 and Rg5, the articular surfaces were not smooth, and part of the cartilage was degenerated and replaced by granulation tissue rich in blood vessels. In Rb1 group, the tissue structure looked slightly abnormal, the surface of the joint was not smooth, and a small amount of effusion could be observed in the joint cavity. For the mice in Rh2 group, except the surface of the joint was not very smooth, no other pathological damage were found. Meanwhile, the overall joint structure of the mice in Rg1 and CK groups were basically normal, with smooth joint surface and normal chondrocyte structure, indicating their good therapeutic effect.

### Effects of ginsenosides on TNF-a and IL-6 protein expression in joint tissues of CIA mice

**Fig. 5:**
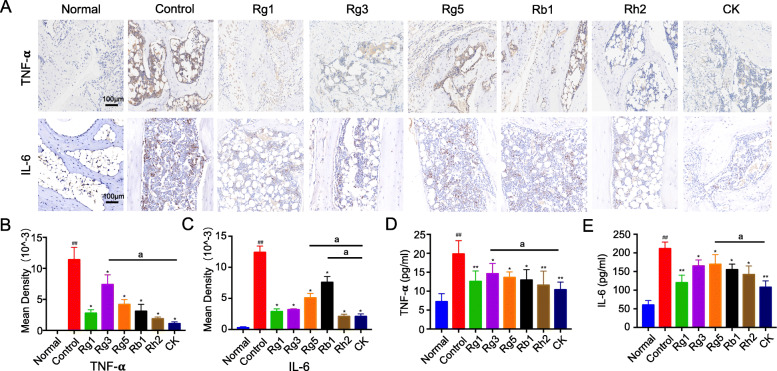
**a** Effects of ginsenosides on TNF-a and IL-6 protein expression in joint tissues of mice (Scale bar = 100 μm). Immunohistochemical staining of osteogenic nuclei is shown to be yellow or brown, indicating positive TNF-α or IL-6. The mean density of TNF-α (**b**) and IL-6 (**c**) protein expression in mouse joints after different treatments. The level of TNF-α (**d**) and IL-6 (**e**) proinflammatory factor in the supernatant of mouse joint after different treatments, respectively. Values were expressed as means ± SD, *n* = 6; (^##^*p* < 0.01 vs. the normal group; * *p* < 0.05 and ** *p* < 0.01 vs the control group; ^a^*p* < 0.05, vs CK group)

From Fig. 5a, it could be found that after immunohistochemical staining, the nuclei of osteoblasts were stained yellow or brown, indicating the positive of TNF-α or IL-6. The results showed that the positive expression of TNF-α and IL-6 in the control group, which was evidently higher than that in the normal group. Although six ginsenosides have different effects on protein expression after drug intervention, they all could decrease the protein expression of TNF-α and IL-6 (Fig. 5b and c), demonstrating that ginsenosides have good inhibitory effect on the expression of TNF-α and IL-6 protein. Among them, CK and Rh2 were proved to be the strongest compounds to attenuate the injure.

In the pathogenesis of RA, a great deal of pro-inflammatory cytokines are overexpressed, so it is important to measure the changes of pro-inflammatory cytokines expression during the treatment [34]. In order to evaluate the inhibitory effect of ginsenosides on proinflammatory cytokines, the expression level of TNF-α and IL-6 in mice joint tissue were further measured with ELISA kit.

As shown in Fig. 5(d and e), the levels of TNF-α and IL-6 were soared in control group (19.84 ± 3.5 pg/ml and 211.50 ± 17.46 pg/ml, respectively), compared with the normal group (7.24 ± 2.15 pg/ml and 60.21 ± 11.92 pg/ml, respectively). After ginsenosides treatment, the content of cytokines was reduced markedly, indicating that ginsenosides could effectively inhibit the development of inflammation, which is consistent with the original hypothesis. And interestingly, among the six drug groups, the ginsenoside CK group had the lowest concentration of TNF-α and IL-6, which were reduced to 10.41 ± 2.0 pg/ml and 108.03 ± 16.86 pg/ml, respectively.

### Effects of ginsenosides on M1-type macrophages and T cells in arthritis

**Fig. 6:**
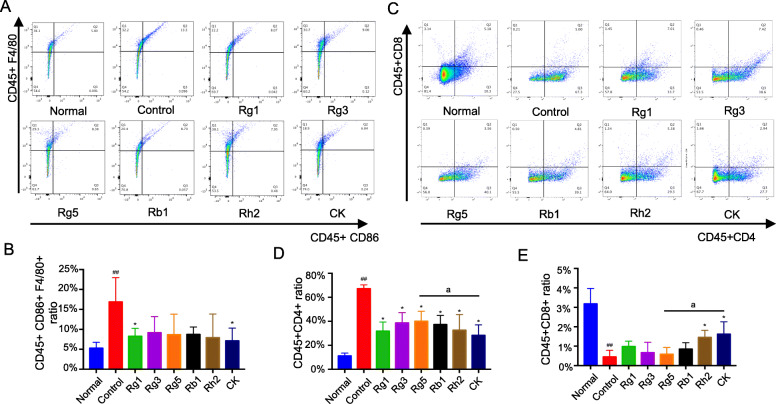
Effects of ginsenosides on M1-type macrophages (**a**, **b**) and T cells (**c**, **d**, **e**) in arthritis. **a** Representative flow scatter plots of CD45 + CD86 and CD45 + F4/80 expressions in the joints of mice in each group. **b** The content of M1-type macrophages (co-expressed CD45 + CD86 and CD45 + F4/80) in the joints of mice in each group. **c** Representative flow scatter plots of CD45 + CD8+ and CD45 + CD4+ expressions in the joints of mice in each group. **d** Expression of CD45 + CD4 + in all groups. **e** Expression of CD45 + CD8 + in all groups. (^*##*^*p* < 0.01, vs normal group; *p < 0.05, vs control group; ^a^*p* < 0.05, vs CK group)

The aggregation of M1-type macrophages at the lesion is related to the severity of RA [35], because they overexpress chemokines and pro-inflammatory factors, such as TNF-α and IL-6. Long-term pro-inflammatory reaction will lead to joint tissue damage, so, reducing the activation of M1-type macrophages is also an effective approach for the treatment of RA. According to the characteristics of M1-type macrophages, CD45+ CD86 and CD45+ F4/80 were co-expressed to label M1-type macrophages, and their quantitative analysis could be performed by flow cytometry [36]. As can be seen from the Fig. 6 (a and b), the CD45+ CD86+ F4/80+ macrophages were enriched in inflamed joints in CIA mice compared with the healthy animals. After ginsenosides intervention, the proportion of CD45+ CD86+ F4/80+ macrophages decreased to some extent. Although the difference between the six groups of ginsenosides was not particularly significant, the content of CD86+ F4/80 + macrophages in CK group was the lowest among the groups.

Besides the influence of macrophages on RA, activated T cells, especially CD4 + T cells [37], also has a non-negligible effect on RA. CD4 + T cells produce proinflammatory cytokines and release a large number of inflammatory mediators, which can cause extensive damage to various systems and organs throughout the body [38]. In addition, we also learned that the frequency of CD8+ T cells in synovial fluid of RA patients was inversely correlated with disease status [39]. A subset of CD8+ T cells, mediated by IL-10 and TGF-β, has suppressor regulatory functions [40]. These cells were produced when attacked by homologous antigens and control inflammation though effector T cells that down-regulate the immune response [41]. Effector CD8+ T cells lead to the death of infected cells by direct lysis or induction of apoptosis [42]. These facts suggest that CD8+ T cells are also involved in regulating inflammation in RA.

Therefore, flow cytometry was used to detect the content of CD45+ CD4+ T cells and CD45+ CD8+ T cells in each group of mice, as shown in Fig. 6(c, d and e). Compared with the healthy mice in normal group, CD45+ CD4+ T cells accumulated significantly in the inflammatory joint of CIA mice, while CD45+ CD8+ T cells were almost absent. Undergo the treatment of various ginsenosides for RA, CD45+ CD4+ content in the diseased mice was significantly decreased, while CD45+ CD8+ content increased obviously. These findings are consistent with the previous that ginsenosides have anti-inflammatory effects. Although the difference among the effect of ginsenosides was not significant, the adjustment effect of ginsenoside CK was also observed to be the strongest.

### Preliminary safety evaluation of ginsenosides

**Fig. 7:**
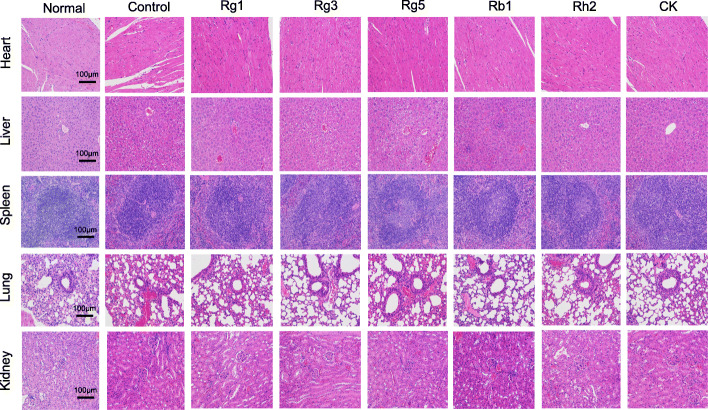
Hematoxylin and Eosin stained sections of the heart, liver, spleen, lung, and kidney of the mice treated with six ginsenosides, PBS and 0.5% Tween was set as normal group and control group, respectively

To assess the safety of ginsenosides, the major organs (including the heart, liver, spleen, lung and kidney) of the mice in each group were collected for H&E staining. As shown in Fig. 7, the cardiomyocytes of healthy mice were clear in structure and the cardiac fibers were long and thin. In the control group, myofibril were unclear and interstitial edema was obvious. The pathological manifestations of the liver provided the information that lobules in normal mice were intact, and the morphology and structure of hepatocytes were radially arranged around the central vein. In the control group, the hepatic cord was disordered and the hepatocytes showed mild or moderate edema. The pathological results of spleen showed that the spleen structure was clear in the normal group and the boundary between white pulp and red pulp was obvious, but the boundary between white pulp and red pulp was fuzzy in the control group. Renal pathology showed that the kidney structure of healthy mice was normal, with a small amount of interstitium and intact epithelial cells of renal tubules. In the control group, glomerular volume was enlarged, epithelial cells of renal tubules were edema and inflammatory cells were infiltrated.

On the contrary, under the guidance of drugs, the deterioration of organ morphology and structure of mice in each drug group was not observed. And the organ morphology tended to normalize, indicating that the toxic and side effect of ginsenosides was very low.

## Discussion

RA is an autoimmune disease characterized by swollen and deformed joints. If the disease were not controlled timely and effectively, it will seriously affects the quality of life, and even may leads patients to disability [43]. At present, the age of the RA onset group is mainly 40–60 years old, but the age of patients group tends to be younger [44]. Therefore, it is urgent to find effective drugs to treat RA. In recent years, the development of biological agents is a major progress in the treatment of RA [45], by blocking the activity of inflammatory mediators that lead to rheumatoid arthritis [46]. However, due to the lack of comprehensive consideration of the disease, the rehabilitation of patients in clinical treatment is not optimistic. The obvious side effects of the drugs increase the physical and mental burden of the patients. Therefore, long-term use of a single biological agent may not be the best way to treat RA.

The traditional plant medicine ginseng is both a medicine and a tonic, so its side effects are not as serious as biological agents. Studies have shown that many kinds of ginsenoside monomers have therapeutic effects on rheumatoid arthritis. Although numerous articles about the pharmacological abilities of natural compounds are published every year, the results from different labs are not consistent, even for same compound. Therefore, it is of significance to compare the effect of different ginsenoside monomers in one lab, find the most effective one and elucidate its action mechanisms for the treatment of RA.

Through in vivo and in vitro experiments in the present study, we could draw a conclusion that six kinds of ginsenoside monomers have a certain therapeutic effect on rheumatoid arthritis. Among them, ginsenoside CK has the best intervention effect, which can not only significantly inhibit the proliferation of RAW264.7 cells and HUVEC cells at low concentration, but also promote the apoptosis of abnormally proliferative cells. Therefore, we speculate that ginsenoside CK has stronger biological activity than other ginsenoside monomers at the same dose. In the collagen-induced mouse experiment, CK also showed the best evidence in substantially reduced swelling, redness, functional impairment of joints and the pathological changes, which have crucial guidance for clinical rheumatoid arthritis patients, because it can significantly improve the quality of life. Meanwhile, the use of ginsenoside CK increased CD8 + T cell to down-regulate the immune response, reduced activated CD4 + T cell number and proinflammatory M1-macrophages resulted in the inhibition of the secretion of proinflammatory cytokine such as TNF-α and IL-6. This result is consistent with the hypothesis that ginsenoside CK relies on T cells to exert its anti-inflammatory effect and alleviate autoimmunity. Combined with the overall experiments, we were pleasantly surprised that ginsenoside Rh2 also showed a certain intervention effect on RA, which provided a new idea for our future study. In addition, we found that when detecting with ELISA method, the expression of TNF-α and IL-6 in the joints of CIA mice was obviously higher than that measured by immunohistochemical method, which may be caused by some non-specific substances retained during the process of sample treatment. Thus, more attention should be paid to eliminate the interference of false positive when using ELISA method in the future.

The pathological mechanisms of RA has not been clearly elucidated, although some anti-RA effects of ginsenosides have been confirmed in the study, more studies needs to be developed to explore the intervention effect of ginsenoside monomers on rheumatoid arthritis from the perspective of signal pathways, and to demonstrate why ginsenoside CK is superior to other ginsenoside in RA treatment.

## Conclusion

The intervention effects of ginsenosides Rg1, Rg3, Rg5, Rb1, Rh2 and CK on rheumatoid arthritis were compared and evaluated in vivo and in vitro for the first time. The results showed that ginsenoside CK has remarkable curative effect on RA with a strong anti-inflammation and immune modulating capabilities, which is expected to be a candidate drug for the treatment of RA.

## Data Availability

All data supporting the conclusions during this study are included in this published article. The datasets used and/or analyzed during the current study are available from the corresponding author on reasonable request.

## Abbreviations

CFA: Complete Freund’s adjuvant
CIA: Collagen-induced arthritis
DMSO: Dimethyl sulfoxide
ELISA: enzyme linked immunosorbent assay
H&E: hematoxylin and eosin
HUVEC cells: Human umbilical vein endothelial cells
IL-6: interleukin 6
LPS: lipopolysaccharide
MTT: 3-[4,5-dimethyl-2-thiazolyl]-2,5-diphenyl-2H-tetrazolium bromide
MTX: Methotrexate
OD: optical density
PI: Propidium iodide
RA: Rheumatoid arthritis
RAW264.7 cells: Mouse leukemia cells of monocyte macrophage
TNF-α: tumor necrosis factor

## References

[1] Smolen JS, Aletaha D, McInnes IB (2016). Rheumatoid arthritis. Lancet.

[2] McInnes IB, Schett G (2017). Pathogenetic insights from the treatment of rheumatoid arthritis. Lancet.

[3] Scott DL, Wolfe F, Huizinga TW (2010). Rheumatoid arthritis. Lancet.

[4] Burmester GR, Pope JE (2017). Novel treatment strategies in rheumatoid arthritis. Lancet.

[5] Aletaha D, Smolen JS (2018). Diagnosis and Management of Rheumatoid Arthritis: a review. JAMA.

[6] Chatzidionysiou K, Emamikia S, Nam J, Ramiro S, Smolen J, van der Heijde D, Dougados M, Bijlsma J, Burmester G, Scholte M, van Vollenhoven R, Landewé R (2017). Efficacy of glucocorticoids, conventional and targeted synthetic disease-modifying antirheumatic drugs: a systematic literature review informing the 2016 update of the EULAR recommendations for the management of rheumatoid arthritis. Ann Rheum Dis.

[7] Smolen JS, Landewe R, Bijlsma J, Burmester G, Chatzidionysiou K, Dougados M, Nam J, Ramiro S, Voshaar M, van Vollenhoven R (2017). EULAR recommendations for the management of rheumatoid arthritis with synthetic and biological disease-modifying antirheumatic drugs: 2016 update. Ann Rheum Dis.

[8] Impellizzeri D, Siracusa R, Cordaro M, Peritore AF, Gugliandolo E, D'Amico R, Fusco R, Crupi R, Rizzarelli E, Cuzzocrea S (2020). Protective effect of a new hyaluronic acid -carnosine conjugate on the modulation of the inflammatory response in mice subjected to collagen-induced arthritis. Biomed Pharmacother.

[9] Suszko A, Obminska-Mrukowicz B (2013). Influence of polysaccharide fractions isolated from Caltha palustris L. on the cellular immune response in collagen-induced arthritis (CIA) in mice. A comparison with methotrexate. J Ethnopharmacol.

[10] Ozen G, Pedro S, Wolfe F, Michaud K (2019). Medications associated with fracture risk in patients with rheumatoid arthritis. Ann Rheum Dis.

[11] Bindu S, Mazumder S, Dey S, Pal C, Goyal M, Alam A, Iqbal MS, Sarkar S, Azhar Siddiqui A, Banerjee C, Bandyopadhyay U (2013). Nonsteroidal anti-inflammatory drug induces proinflammatory damage in gastric mucosa through NF-kappaB activation and neutrophil infiltration: anti-inflammatory role of heme oxygenase-1 against nonsteroidal anti-inflammatory drug. Free Radic Biol Med.

[12] Strehl C, Bijlsma JW, de Wit M, Boers M, Caeyers N, Cutolo M, Dasgupta B, Dixon WG, Geenen R, Huizinga TW (2016). Defining conditions where long-term glucocorticoid treatment has an acceptably low level of harm to facilitate implementation of existing recommendations: viewpoints from an EULAR task force. Ann Rheum Dis.

[13] Ramaswami R, Stebbing J (2013). Ginseng: panacea among herbal remedies?. Lancet Oncol.

[14] Cheng Z, Zhang M, Ling C, Zhu Y, Ren H, Hong C, Qin J, Liu T, Wang J. Neuroprotective Effects of Ginsenosides against Cerebral Ischemia. Molecules. 2019;24(6):1102. 10.3390/molecules24061102. 10.3390/molecules24061102 PMC6471240 30897756

[15] Kim JH (2018). Pharmacological and medical applications of Panax ginseng and ginsenosides: a review for use in cardiovascular diseases. J Ginseng Res.

[16] Kim JW, Kim JM, Kim SK, Kim YM, Choi JS (2018). Protective effect of ginseng on salivary dysfunction following radioiodine therapy in a mouse model. Thyroid.

[17] Yi YS (2019). Roles of ginsenosides in inflammasome activation. J Ginseng Res.

[18] Yao F, Xue Q, Li K, Cao X, Sun L, Liu Y. Phenolic Compounds and Ginsenosides in Ginseng Shoots and Their Antioxidant and Anti-Inflammatory Capacities in LPS-Induced RAW264.7 Mouse Macrophages. Int J Mol Sci. 2019;20(12):2951. 10.3390/ijms20122951. 10.3390/ijms20122951 PMC6627944 31212928

[19] Yang WS, Yi YS, Kim D, Kim MH, Park JG, Kim E, Lee SY, Yoon K, Kim JH, Park J, Cho JY (2017). Nuclear factor kappa-B- and activator protein-1-mediated immunostimulatory activity of compound K in monocytes and macrophages. J Ginseng Res.

[20] Chen J, Wu H, Wang Q, Chang Y, Liu K, Song S, Yuan P, Fu J, Sun W, Huang Q, Liu L, Wu Y, Zhang Y, Zhou A, Wei W (2014). Ginsenoside metabolite compound k alleviates adjuvant-induced arthritis by suppressing T cell activation. Inflammation.

[21] Chen J, Wang Q, Wu H, Liu K, Wu Y, Chang Y, Wei W (2016). The ginsenoside metabolite compound K exerts its anti-inflammatory activity by downregulating memory B cell in adjuvant-induced arthritis. Pharm Biol.

[22] Kim HA, Kim S, Chang SH, Hwang HJ, Choi YN (2007). Anti-arthritic effect of ginsenoside Rb1 on collagen induced arthritis in mice. Int Immunopharmacol.

[23] Wang Y, Chen J, Luo X, Zhang Y, Si M, Wu H, Yan C, Wei W (2016). Ginsenoside metabolite compound K exerts joint-protective effect by interfering with synoviocyte function mediated by TNF-alpha and tumor necrosis factor receptor type 2. Eur J Pharmacol.

[24] Zhang L, Zhu M, Li M, Du Y, Duan S, Huang Y, Lu Y, Zhang J, Wang T, Fu F (2017). Ginsenoside Rg1 attenuates adjuvant-induced arthritis in rats via modulation of PPAR-gamma/NF-kappaB signal pathway. Oncotarget.

[25] Choi YS, Kang EH, Lee EY, Gong HS, Kang HS, Shin K, Lee EB, Song YW, Lee YJ (2013). Joint-protective effects of compound K, a major ginsenoside metabolite, in rheumatoid arthritis: in vitro evidence. Rheumatol Int.

[26] Chen J, Wu H, Wang Q, Chang Y, Liu K, Wei W (2015). Ginsenoside metabolite compound K suppresses T-cell priming via modulation of dendritic cell trafficking and costimulatory signals, resulting in alleviation of collagen-induced arthritis. J Pharmacol Exp Ther.

[27] Liu KK, Wang QT, Yang SM, Chen JY, Wu HX, Wei W (2014). Ginsenoside compound K suppresses the abnormal activation of T lymphocytes in mice with collagen-induced arthritis. Acta Pharmacol Sin.

[28] Liang Y, Wu J, Li Y, Li J, Ouyang Y, He Z, Zhao S (2015). Enhancement of ginsenoside biosynthesis and secretion by tween 80 in Panax ginseng hairy roots. Biotechnol Appl Biochem.

[29] Brand DD, Latham KA, Rosloniec EF (2007). Collagen-induced arthritis. Nat Protoc.

[30] Ren H, He Y, Liang J, Cheng Z, Zhang M, Zhu Y, Hong C, Qin J, Xu X, Wang J (2019). Role of liposome size, surface charge, and PEGylation on rheumatoid arthritis targeting therapy. ACS Appl Mater Interfaces.

[31] Korb-Pap A, Stratis A, Muhlenberg K, Niederreiter B, Hayer S, Echtermeyer F, Stange R, Zwerina J, Pap T, Pavenstadt H (2012). Early structural changes in cartilage and bone are required for the attachment and invasion of inflamed synovial tissue during destructive inflammatory arthritis. Ann Rheum Dis.

[32] Kennedy A, Fearon U, Veale DJ, Godson C (2011). Macrophages in synovial inflammation. Front Immunol.

[33] Bhattaram P, Chandrasekharan U (2017). The joint synovium: a critical determinant of articular cartilage fate in inflammatory joint diseases. Semin Cell Dev Biol.

[34] Feldmann M, Maini RN (2003). Lasker clinical medical research award. TNF defined as a therapeutic target for rheumatoid arthritis and other autoimmune diseases. Nat Med.

[35] Kinne RW, Brauer R, Stuhlmuller B, Palombo-Kinne E, Burmester GR (2000). Macrophages in rheumatoid arthritis. Arthritis Res.

[36] Put S, Westhovens R, Lahoutte T, Matthys P (2014). Molecular imaging of rheumatoid arthritis: emerging markers, tools, and techniques. Arthritis Res Ther.

[37] Fonseka CY, Rao DA, Raychaudhuri S (2017). Leveraging blood and tissue CD4+ T cell heterogeneity at the single cell level to identify mechanisms of disease in rheumatoid arthritis. Curr Opin Immunol.

[38] Fonseka CY, Rao DA, Teslovich NC, Korsunsky I, Hannes SK, Slowikowski K, Gurish MF, Donlin LT, Lederer JA, Weinblatt ME, et al. Mixed-effects association of single cells identifies an expanded effector CD4(+) T cell subset in rheumatoid arthritis. Sci Transl Med. 2018;10(463):eaaq0305. 10.1126/scitranslmed.aaq0305. 10.1126/scitranslmed.aaq0305 PMC6448773 30333237

[39] Cho BA, Sim JH, Park JA, Kim HW, Yoo WH, Lee SH, Lee DS, Kang JS, Hwang YI, Lee WJ, Kang I, Lee EB, Kim HR (2012). Characterization of effector memory CD8+ T cells in the synovial fluid of rheumatoid arthritis. J Clin Immunol.

[40] Wang YM, Alexander SI (2009). CD8 regulatory T cells: what's old is now new. Immunol Cell Biol.

[41] Hu D, Ikizawa K, Lu L, Sanchirico ME, Shinohara ML, Cantor H (2004). Analysis of regulatory CD8 T cells in Qa-1-deficient mice. Nat Immunol.

[42] Carvalheiro H, da Silva JA, Souto-Carneiro MM (2013). Potential roles for CD8(+) T cells in rheumatoid arthritis. Autoimmun Rev.

[43] Safiri S, Kolahi AA, Hoy D, Smith E, Bettampadi D, Mansournia MA, Almasi-Hashiani A, Ashrafi-Asgarabad A, Moradi-Lakeh M, Qorbani M, Collins G, Woolf AD, March L, Cross M (2019). Global, regional and national burden of rheumatoid arthritis 1990-2017: a systematic analysis of the global burden of disease study 2017. Ann Rheum Dis.

[44] Curtis JR, Greenberg JD, Harrold LR, Kremer JM, Palmer JL (2018). Influence of obesity, age, and comorbidities on the multi-biomarker disease activity test in rheumatoid arthritis. Semin Arthritis Rheum.

[45] Chaudhari K, Rizvi S, Syed BA (2016). Rheumatoid arthritis: current and future trends. Nat Rev Drug Discov.

[46] Ramiro S, Sepriano A, Chatzidionysiou K, Nam JL, Smolen JS, van der Heijde D, Dougados M, van Vollenhoven R, Bijlsma JW, Burmester GR, Scholte-Voshaar M, Falzon L, Landewé RBM (2017). Safety of synthetic and biological DMARDs: a systematic literature review informing the 2016 update of the EULAR recommendations for management of rheumatoid arthritis. Ann Rheum Dis.

